# Hyperlipidemia Induced Pathological Changes with no Effect in Biomechanical Properties in the Achilles Tendon of Young Swine

**DOI:** 10.26502/josm.511500144

**Published:** 2024-04-22

**Authors:** Merlin Rajesh Lal L.P, Devendra K. Agrawal

**Affiliations:** Department of Translational Research, College of Osteopathic Medicine of the Pacific, Western University of Health Sciences, Pomona, California USA

**Keywords:** Achilles tendon, Biomechanics, Dynamic modulus, Hyperlipidemia, Mechanical properties

## Abstract

Hyperlipidemia is linked to atherosclerosis and various diseases. Its strong association with Achilles tendinopathies and xanthomas affects tendon properties through lipid deposition in tendon tissue. We examined the impact of hyperlipidemia on the biomechanical properties of the swine Achilles tendons. Swines were fed a high-cholesterol-high-fat diet to induce hyperlipidemia, and their Achilles tendons were collected and examined for biomechanical properties. The ultimate tensile strength, modulus of elasticity and viscoelastic properties did not exhibit significant differences between hyperlipidemic and control swines. H&E and pentachrome staining revealed extracellular matrix (ECM) disorganization and cellular infiltration in the hyperlipidemic swines, highlighting a marked difference between the control and hyperlipidemic groups. These results suggest hyperlipidemia in young swines alters the tendon composition and may contribute to weak biomechanical properties with time.

## 1. Introduction

Hyperlipidemia is recognized as the primary cause of atherosclerosis. Recent reports highlight the emergence of tendinopathy associated with hyperlipidemia [1]. Hypercholesterolemia is known to induce tendinous abnormalities, including thickening and the formation of tendinous xanthomas, where lipids accumulate in the tendons [2]. The Achilles tendon, a crucial energy-storing tendon, is frequently affected by this [3]. The accumulation of lipids and tendinopathies disrupts tendon biomechanics. A recent study has reported Achilles tendon thickening to an increased risk of cardiovascular disease. Numerous studies indicate that elevated low-density lipoprotein (LDL) cholesterol levels may contribute to tendinopathy by depositing lipids in the tendon’s extracellular matrix (ECM) and can compromise the mechanical properties of tendons and ligaments under hyperlipidemic conditions [4].

The ECM components of Achilles tendons play a crucial role in managing the high forces experienced during physical activities by transferring loads from muscles to bones. The extension of tendons is facilitated by the sliding movement of collagen fibrils, from a hierarchical arrangement of collagen and other ECM components within the interfascicular matrix [5,6]. Hence, researchers are keenly interested in understanding the association between hyperlipidemia and changes in Achilles tendons to explore new avenues for treatment. In our previous studies on swine models, we reported that hyperlipidemia induced pathological changes in tendon organization, extracellular matrix (ECM), and cellularity [7] along with early changes in the mechanical properties of the infraspinatus tendon [8,9]. Our current focus is on studying the changes in the biomechanical properties of the Achilles tendon in swine under a hyperlipidemic diet.

## 2. Materials and Methods

### 2.1 Animals and tendon Tissue Collection and Preparation

The Institutional Animal Care and Use Committee (IACUC) of Western University of Health Sciences, Pomona, CA, USA approved the experimental research protocol (R22IACUC034).

Female Yucatan miniswine (Sus scrofa) weighing 25–30 kg, were purchased from Premier Bioresources, Ramona, CA, USA. Swines were either fed with regular pig diet (Group-1) or with high-cholesterol-high-fat diet (Group-2) to develop hyperlipidemia. The animals were fed twice every day with 12/12 hours of light-dark cycle. At 40 weeks, swines were sacrificed, and the Achilles tendon tissues were collected and stored at −80°C until testing [7,9,10]. On the day of testing the tendon tissues were thawed using a two-step protocol: (i) 4 h at 4°C, and (ii) 2 h at room temperature [11]. Two equal pieces of approximately 55 mm in length were dissected using scalpel blade and used for tensile testing and Dynamic mechanical analysis (DMA). The tissues were maintained at 4°C to preserve the freshness until testing.

### 2.2 Tensile strength and strain

Tensile testing was carried out on 4 tendons from control animals and 6 tendons from hyperlipidemic animals. The length, width, and thickness of tendons were measured using a digital vernier caliper and the smaller dimensions was utilized to calculate the cross-sectional area. Tendons were secured between sandpaper and mounted using tension grips on a TA Electroforce 3300 (TA Instruments, New Castle, DE, USA) equipped with a 1000 N load cell. The gauge length was set at 30 mm. The samples were preconditioned for 10 cycles at a frequency of 1.0 Hz between 0 and 10 N under load control [12]. Following preconditioning, tendon tissue samples were ramped to failure at a crosshead speed of 100 μm/s while continuously recording force and displacement. Failure was defined as the decrease in load below 20% of the maximum load. Engineering stress (σ) and engineering strain (ε) were determined, and a stress-strain curve was plotted. The modulus of elasticity (E) was obtained from the slope of the linear region of the stress-strain curve. Ultimate tensile strength (UTS) and strain at failure were also calculated [13,14].

### 2.3 Dynamic mechanical analysis

Dynamic Mechanical Analysis (DMA) was conducted on four tendons from Group-1 swines (control) and six tendons from Group-2 swines subjected to a hyperlipidemic diet. Following measurements of length, width, and thickness using a digital vernier caliper, the tendons were secured between sandpaper and mounted using tension grips onto the TA Electroforce 3300 (TA Instruments, New Castle, DE, USA) equipped with a 1000 N load cell. An initial load of 2.0 N was applied to eliminate slack, and the gauge length was recorded. The tendon tissues underwent preconditioning with 10 cycles at a frequency of 1.0 Hz between 0 and 10 N under load control. Sinusoidal loading between 10 N and 20 N was applied, and two frequency sweeps were performed: (i) from 0.2 Hz to 2.0 Hz with an increase in frequency of 0.2 Hz until 2 Hz, and (ii) from 1 Hz to 41 Hz with an increase in frequency of 10 Hz until 41 Hz. Throughout the testing, the samples were immersed in PBS at 37°C. The machine calculated the viscoelastic properties, including dynamic modulus (E*), storage modulus (E’), loss modulus (E”), and damping ability (Tan δ) and the values were recorded [11,12].

### 2.4 Water content

The water content of the tendon core (10 mm length, 3–4 mm width and thickness) was calculated based on the weight of tendon tissues before ($W_{wet}$) and after drying ($W_{dry}$) for 72 hours at 40°C. The samples were weighed using analytical balance with the resolution of 0.01 mg [8]. The water content was calculated using the below formula:

$$Water\;content=\frac{W_{wet}\;-\;W_{dry}}{W_{wet}}\;\times 100$$

### 2.5 Histology

Tendon tissue samples were fixed with 10% buffered formalin for 72 hours, processed, embedded in paraffin, and 7 μm thick sections were cut. The sections were stained with hematoxylin and eosin (H&E), pentachrome stain and analyzed for cellularity, ECM organization, and tendon alignment.

### 2.6 Statistical analysis

Statistical analyses for tensile testing, modulus of elasticity, failure strain %, and water content were conducted using unpaired Student t-tests. Dynamic mechanical analysis results were calculated using a two-way analysis of variance (ANOVA) using GraphPad Prism 9.5.1 software. A p-value of < 0.05 was considered statistically significant.

## 3. Results

### 3.1 Tensile strength

**Figure 1:**
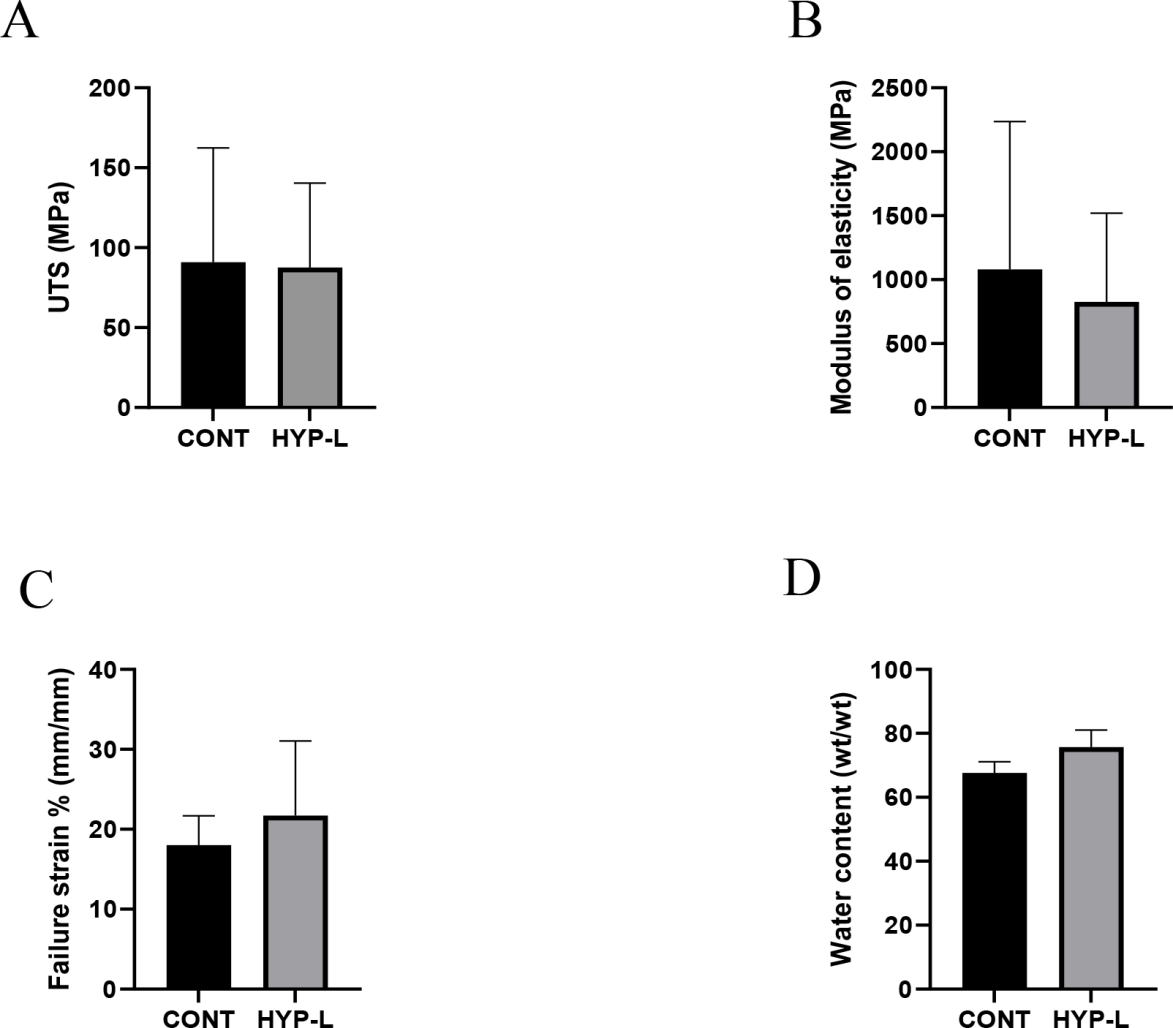
Mechanical properties of Achilles tendon tissues: (A) Ultimate tensile strength, (B) Modulus of elasticity, (C) Failure strain %, and (D) Water content. CONT indicates the group of animals that received normal diet (Group 1), Hyp-L indicates the group of animals that received high-cholesterol-high-fat diet (Group 2). Values are shown as mean ± SD; n=4–6). * Indicates significant difference (p<0.05, student-t test).

The ultimate tensile strength (UTS), failure strain percentage, and the modulus of elasticity (E) are shown in Figure 1. Even though there was a decrease in the UTS and increase in elastic modulus in the hyperlipidemia group of swines, the difference is not statistically significant (Figures 1A and 1B). The failure strain percentages did not have any significant difference between groups (Figure 1C). The water content of tendons from the control group (67.62 ± 3.47 %) and the hyperlipidemic group (74.02 ± 5.72 %) were not statistically significant (Figure 1D).

### 3.2 Dynamic Modulus Analysis (DMA)

**Figure 2:**
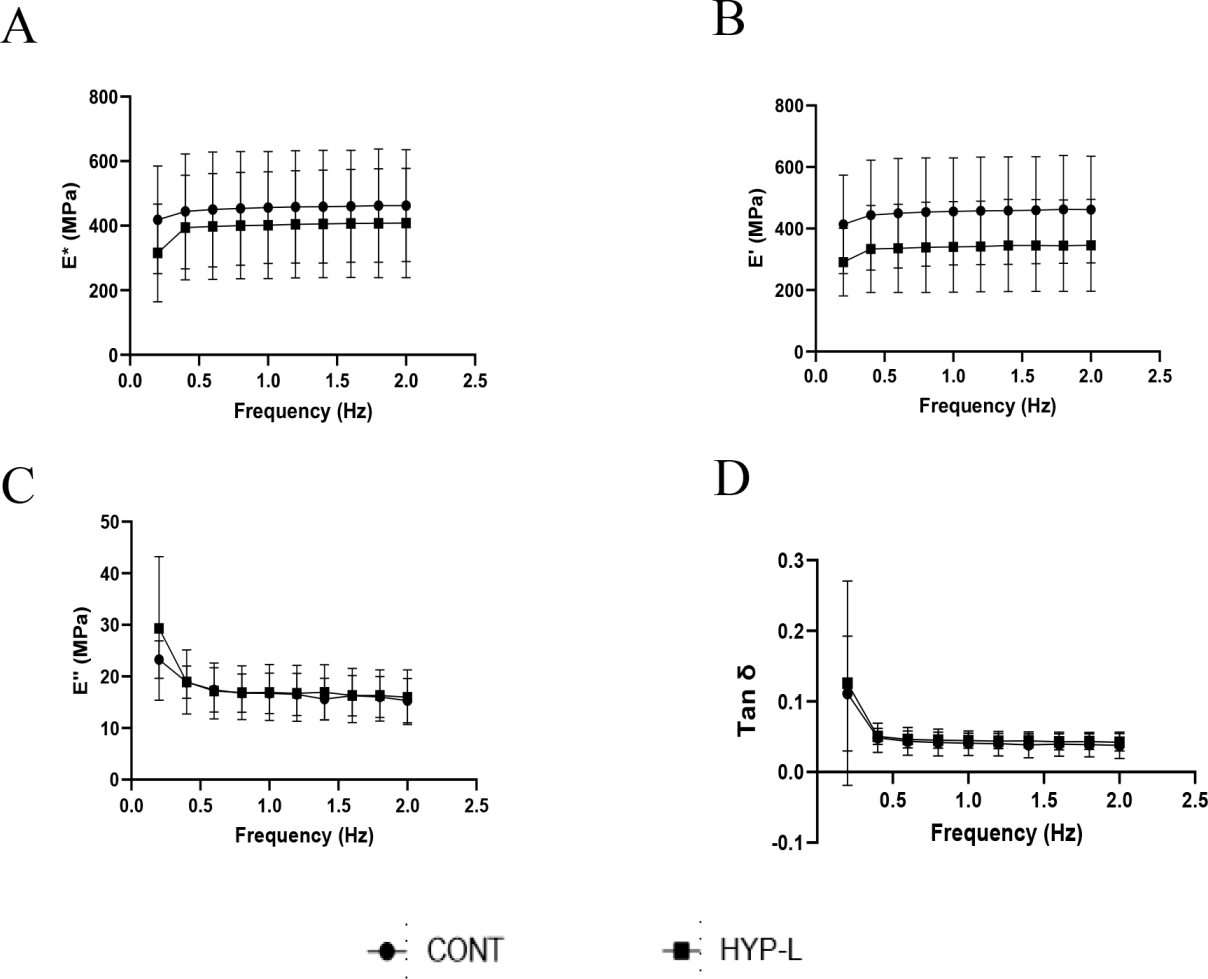
Viscoelastic properties of Achilles tendon tissues at low frequencies: (A) dynamic modulus (E*), (B) storage modulus (E’), (C) loss modulus (E”) and (D) damping ability (Tan δ). CONT indicates the group of animals that received normal diet, Hyp-L indicates the group of animals that received high-cholesterol-high-fat diet. Values are shown as mean ± SD; n=4–6).

**Figure 3:**
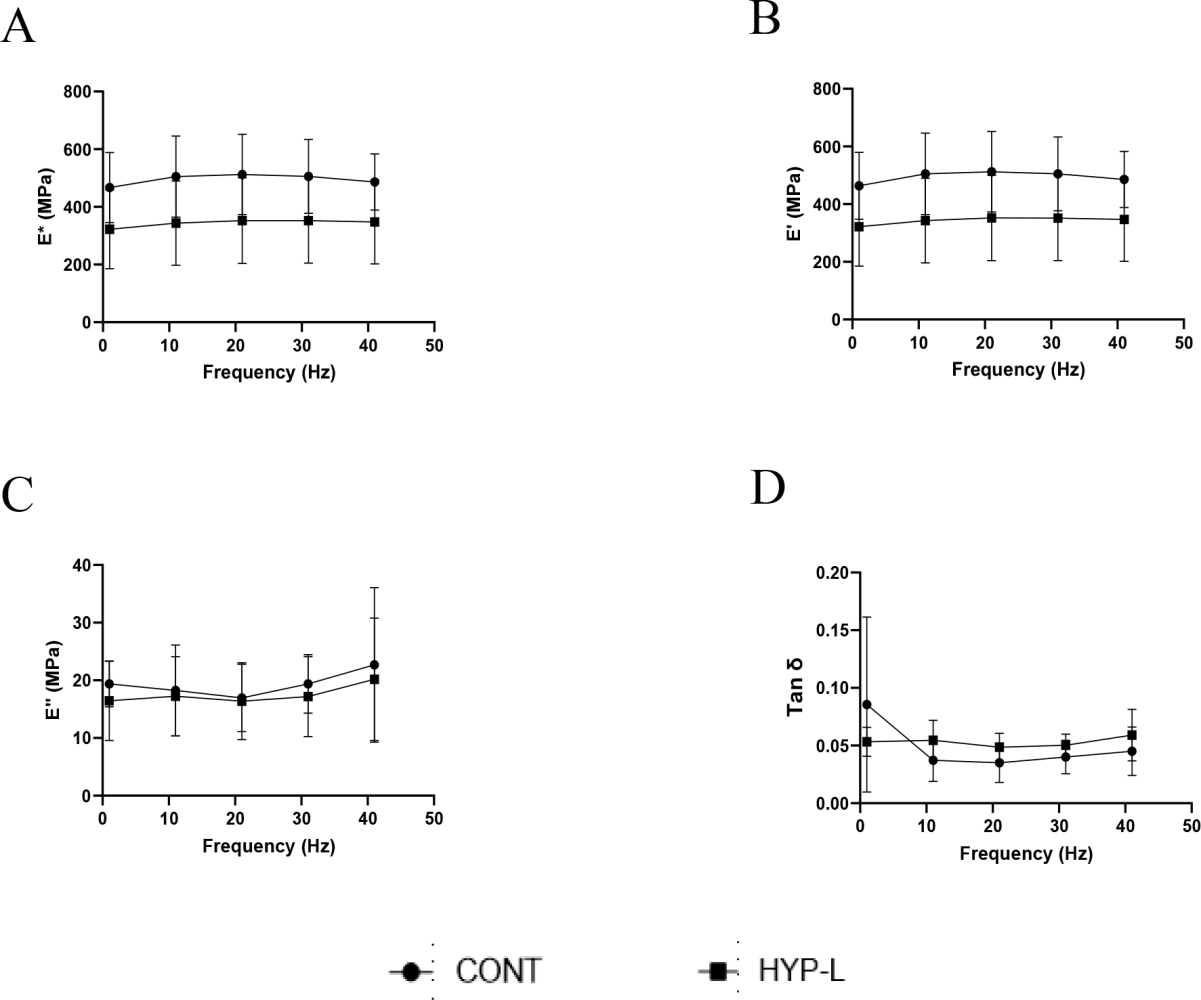
Viscoelastic properties of the tendon tissues at higher frequencies: (A) dynamic modulus (E*), (B) storage modulus (E’), (C) loss modulus (E”) and (D) damping ability (Tan δ). CONT indicates the group of animals that received normal diet, Hyp-L indicates the group of animals that received high-cholesterol-high-fat diet. Values are shown as mean ± SD, n=4–6).

The viscoelastic properties recorded from the dynamic mechanical analysis at low frequencies are shown in Figure 2. The dynamic modulus (Figure 2A) and storage modulus (Figure 2B) increased with increase in frequency, but were not significantly different with each other. The loss modulus (Figure 2C) and damping ability (Tan δ) (Figure 2D) were not significantly different among all the tested frequencies. The differences in the dynamic modulus and storage modulus between control group and hyperlipidemic group were not significant at respective frequencies tested, at low (0.2–2 Hz) (Figure 2) and higher tested (Figure 3).

### 3.2 Histology

**Figure 4:**
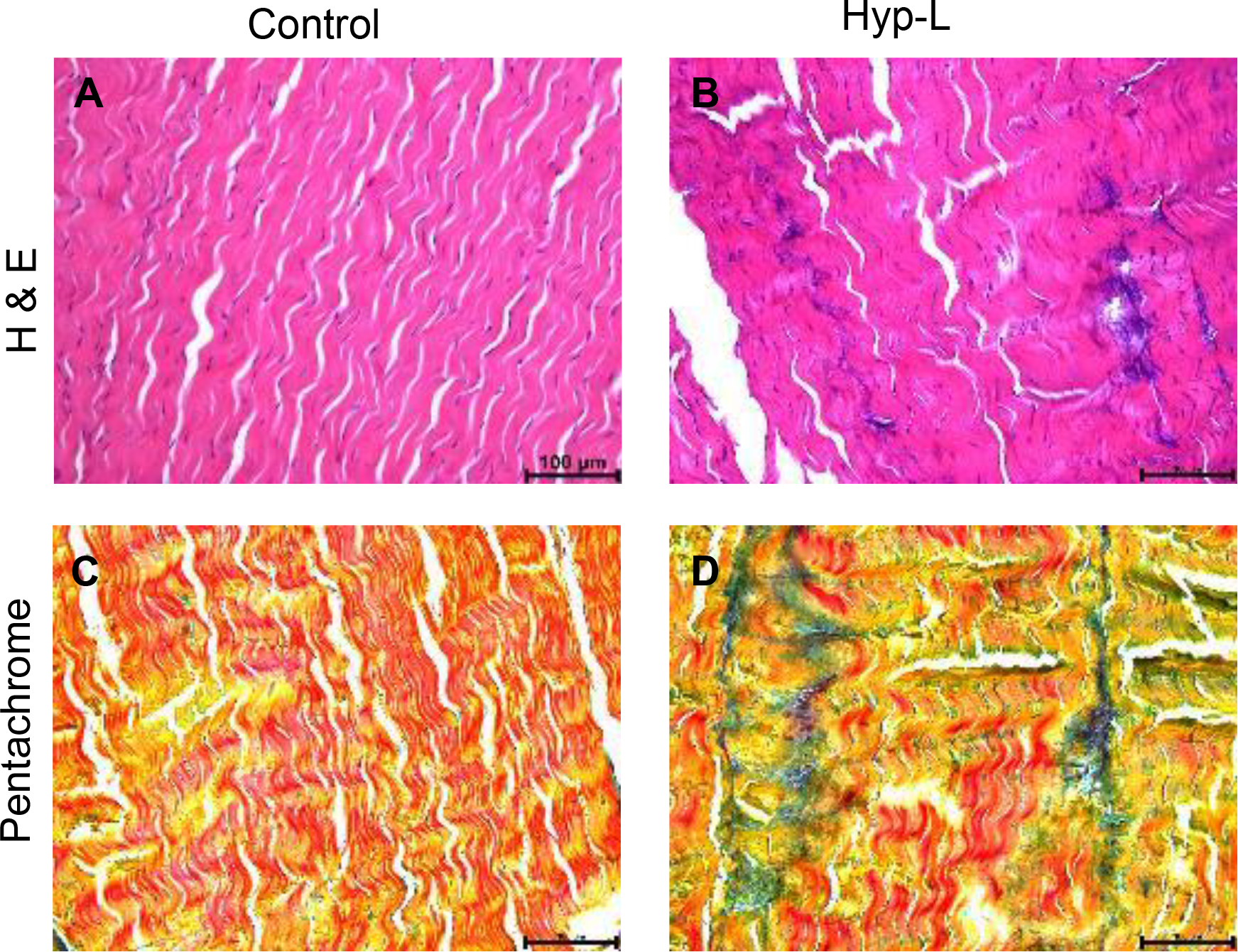
Representative image of tendon H&E staining (A, B) and pentachrome staining (C, D). The left panel represents the control group. Right panel (B and D) represents hyperlipidemic group. Control (left panel) indicates the group of animals that received normal diet (group 1), Hyp-L (right panel) indicates the group of animals that received high-cholesterol-high-fat diet (Group 2). The images were acquired in 20× magnification.

The representative image of H&E staining of the tendon tissue is shown in Figures 4A and 4B. The control group were presented with matured tenocytes with elongated nuclei in a well-organized, aligned fibers of extra cellular matrix (ECM). The hyperlipidemic group had disorganized ECM and infiltered with proliferating cells like tenoblast and inflammatory cells. Representative images of pentachrome staining are shown in Figures 4C and 4D, which revealed that Achilles tendon ECM of the hyperlipidemic swines were disorganized and low staining for collagen compared to the control swines. Moreover, there is an increased staining for elastic fibers (Figure 4D) in the hyperlipidemic swines compared to the control swines that received normal diet.

## 4. Discussion

Hypertension, cardiovascular diseases, and diabetes are strongly linked to hyperlipidemia [15]. Hyperlipidemia is known to deposit lipids that integrate into the extracellular matrix (ECM) of tendon tissues, thereby compromising their properties [16]. Our focus was to investigate the impact of hyperlipidemia on the biomechanical characteristics of the Achilles tendon, recognized as the largest and energy-storing tendon [3]. Recent findings suggest that thickening of the Achilles tendon poses a risk for cardiovascular disease and affect the biomechanical properties of the tendon [17–19]. In our study, we observed no significant differences in the ultimate tensile strength of the Achilles tendon. The modulus of elasticity was lower in hyperlipidemic swine compared to control swine, but these differences were not statistically significant due to observed variations among swine subjects.

Hyperlipidemia has been reported to decrease the modulus of elasticity in infraspinatus tendons and biceps tendons in swine [8] which was in contrast with previous studies on supraspinatus tendons in hyperlipidemic rat and mouse models [20,21]. The linear range of motion of tendon is the result of collagen fibril sliding to each other, where modulus of elasticity is obtained from the stress-strain curve. The decrease in modulus of elasticity indicates the tendon is inferior and will deform more for the same amount of load and hence increase the failure strain before break in tensile testing [22]. We observed a similar trend of lowered modulus of elasticity and increased failure strain % in hyperlipidemic swines in our study, even though the values were not statistically significant.

The viscoelastic properties displayed no significant difference in both lower frequencies and at higher frequencies. Though dynamic modulus and storage modulus were not significantly different between the control swines and hyperlipidemic swines, the hyperlipidemic swines had consistently lower values than the control swines. These are initial changes due to hyperlipidemic condition [8,16,23]. We did expect little changes in the viscoelastic properties, because the swines in our study were young (40 weeks) and active. The osmotic balance of the tissue during testing is very important, since it can affect testing results. Previous studies on human cadaveric iliotibial band tendon have shown that as the osmotic pressure increases the water content decreased, conversely the modulus of elasticity increased with increase in osmotic pressure [9,24]. To have the testing environment constant we performed dynamic mechanical analysis in PBS bath, hence the differences we observed are inherent to tendon tissues.

The difference in water content of the tendon tissues of hyperlipidemic group (74.02±5.72 w/w) and control swines (67.62±3.47 w/w) were not significant. Hyperlipidemia increases the proteoglycan content, vascularity and hence increases the water content in tendons [25]. Previous studies state that hyperlipidemia alters the native ECM composition of the tendons and hence affecting the biomechanical properties of the tendons [26]. The modulus of elasticity, failure strain and water content display a characteristically weak tendon, but these are early stages of weakening and differences are not pronounced in young swines [8]. Mechanical properties of tendon tissues decrease with age and lack of physical activities, while hyperlipidemia could surge the process [27].

We observed intercalation of lipids and disorganization of the tendon ECM with infiltrating inflammatory cell in the tendon tissues of the hyperlipidemic swines. This is observed in injured tendons and actively remodeling tendons [28,29]. While fat get deposited in the tendon tissue, presence of inflammatory cells is reported, and integrity of tendon is disrupted, but the mechanism of initial trigger is still unclear [30–33]. With time and further addition of lipids, it invites a cascade of events that unfolds the inflammation and further weakens the tendons by altering tendon ECM and collagen fibrils. With the lipid accumulation and associated inflammation, the collagen content gets reduced [34,35] and shall contribute to weak biomechanical properties.

In this study the swines were young and active, hence we could rule out the age-related change in tendons, but the changes in tendon tissue ECM is evident in the hyperlipidemic swines. These are early changes in the tendon tissues which shall further contribute to the deteriorated biomechanical properties. The break point beyond which the pathological changes that contribute to altered biomechanical properties are still unclear. Further, detailed studies on the factors that trigger the accumulation of lipids and initial inflammatory trigger need to be studied in detail coupled with long term studies to understand the pathophysiology and to develop new targets for treatments.

## 5. Conclusion

This study reports early insights on the biomechanical properties of Achilles tendon of swine under hyperlipidemic diet. The ECM disorganization and inflammatory cell infiltration were observed in the tendon of hyperlipidemic swines but have not contributed to the biomechanical properties. However long-term studies are necessary to understand the breakout of differences in tendon biomechanics with physical activities, age and hyperlipidemia.

## 6. Limitations

The tendon tissues at the time of tissue collection were relatively young and the sample size was small. Also, the mechanical properties vary with variation in physical activities. The measurement of extension was calculated from the crosshead displacement of clamps to compute the strain values. Measurement with video extensometer would have provided better information on the parameters. Moreover, the variation in the physical activities warrants further investigation in detail as the swine were allowed to walk freely in the pen.
